# Anterior Hyaloid Staining With Trypan Blue During Phacoemulsification

**DOI:** 10.7759/cureus.96135

**Published:** 2025-11-05

**Authors:** Dimitrios Tsouris, Olga Tsouri, Achilleas Mandalos

**Affiliations:** 1 Ophthalmology, General Hospital of Larissa, Larissa, GRC; 2 Ophthalmology, Aristotle University of Thessaloniki, Thessaloniki, GRC; 3 Ophthalmology, Karditsa District General Hospital, Karditsa, GRC

**Keywords:** blunt ocular trauma, cataract, cataract surgery complications, sports-related eye injuries, trypan blue

## Abstract

A semi-professional football player diagnosed with a left eye cataract was scheduled for phacoemulsification. Extensive preoperative assessment identified no risk factors. A trypan blue-assisted capsulorhexis was planned. Following anterior chamber wash-out with a balanced salt solution and intracameral application of an ophthalmic viscosurgical device, spontaneous dye migration into the vitreous cavity was noticed. We would like to present this rare complication in the absence of evident zonular pathology and its immediate management.

## Introduction

Cataract represents one of the leading causes of visual impairment worldwide. Phacoemulsification is established as the mainstay method for cataract extraction. Through a sequence of precisely executed surgical steps, a cataractous crystalline lens is replaced by an artificial one, conferring an immediate positive effect on the patient's visual function and quality of life.

Undeniably, continuous curvilinear capsulorhexis represents one of the most crucial steps of the procedure. Capsular staining using trypan blue (TB) is recognized as a safe method that improves the visualization of the anterior capsule and thus facilitates this critical step [1]. Zonular integrity in the vast majority of cases precludes dye migration into the vitreous cavity, a rare phenomenon that is, however, encountered in cases of preoperatively identified zonular compromise, such as in traumatic cataracts, in the presence of pseudoexfoliation, uveitis, or high myopia, and in cases with a previous history of ophthalmic surgical procedures. Dye diffusion into the vitreous impairs the visualization, rendering the procedure quite challenging. In addition, it raises concerns of possible retinal toxicity [2].

In this case report, we would like to present the spontaneous TB dye migration into the anterior hyaloid during phacoemulsification of a young soccer athlete without clinically evident phacodonesis and in the absence of any of the aforementioned risk factors that could compromise the zonular apparatus. The rationale of our management, in addition to the final outcome, is also discussed. 

## Case presentation

A 32-year-old male semi-professional soccer (football) player presented in May 2024 with a left eye cataract affecting his vision. Detailed preoperative assessment did not identify clinically evident phacodonesis or zonular dehiscence. Subsequently, he was scheduled for left phacoemulsification with enhanced depth of focus toric intraocular lens (IOL) implant under topical anesthesia. A continuous curvilinear capsulorhexis with TB 0.6 mg/ml ophthalmic solution assistance was planned.

Following partial fill of the anterior chamber with air, a small volume of approximately 0.05 ml of TB was intracamerally administered without excessive pressure, through the 20G sideport incision. Interestingly, during this step, both TB dye-stained aqueous humor and air escaped from the sideport incision, preventing anterior chamber over-inflation. Subsequently, through the main limbal incision, the residual TB dye was irrigated with a balanced salt solution (BSS).

During reformation of the anterior chamber with high-molecular-weight sodium hyaluronate 1%, inadvertent migration of the TB into the anterior vitreous was noticed, progressively obscuring the fundal red reflex and rendering the subsequent capsulorhexis surgical step challenging.

However, the case proceeded without additional complications encountered. Interestingly, throughout the procedure, the crystalline lens remained centered, without evident phacodonesis. Neither during nucleofractis nor during cortical aspiration did the zonules demonstrate signs of impairment.

Upon completion of phacoemulsification, two options were presented to the patient: either a conservative approach of close monitoring of TB dye gradual absorption or an immediate 25G pars plana vitrectomy with a view to prevent the dye-induced visual impairment and to eliminate the slight risk of possible retinal toxicity. The patient chose the latter option, embarking on pars plana vitrectomy, following which the case was concluded with IOL implantation (Video 1).

**Video 1:** Spontaneous trypan blue migration into the anterior vitreous through intact zonules during routine phacoemulsification

**Figure 1:**
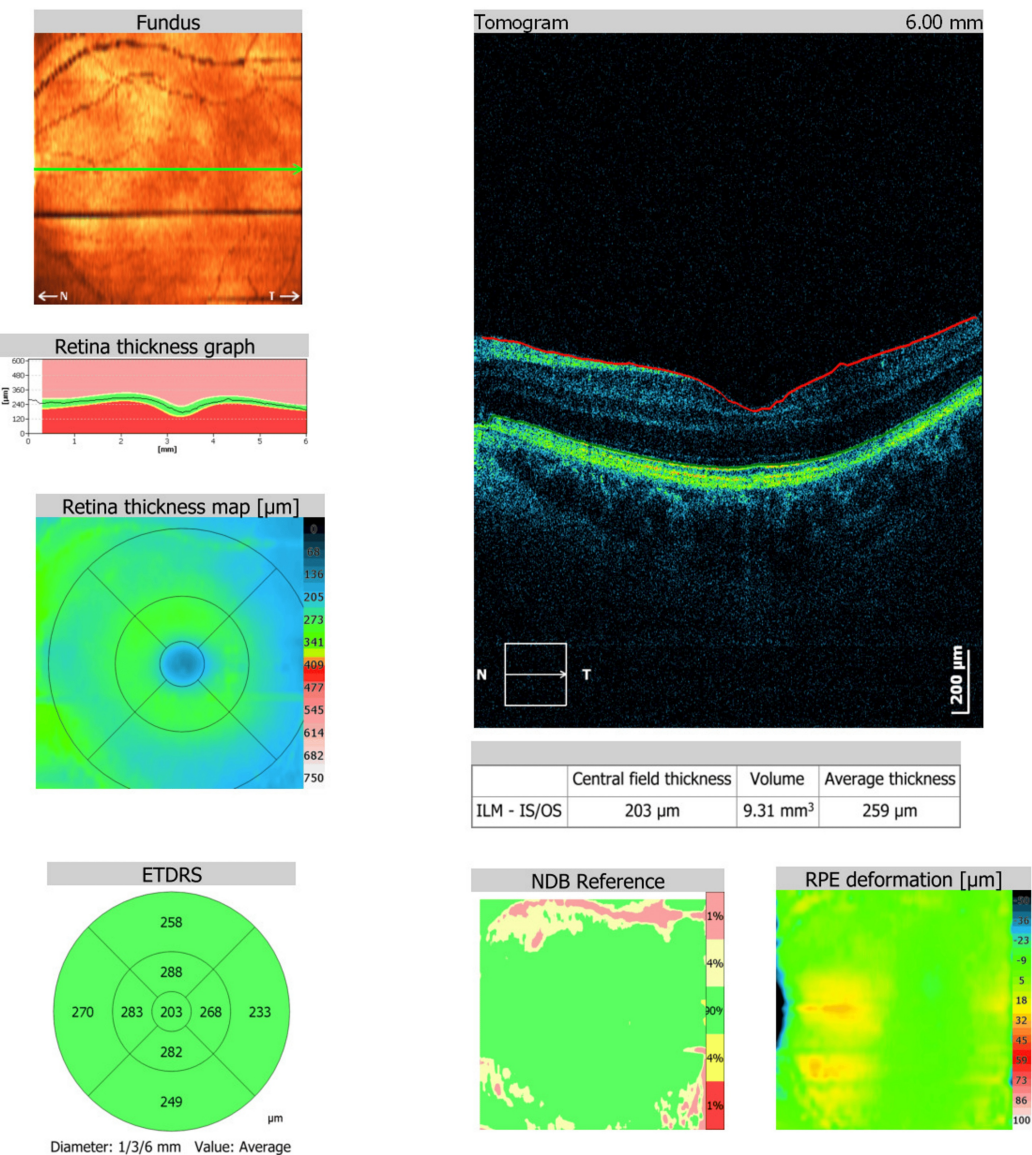
Postoperative spectral domain optical coherence tomography: normal foveal contour NDB: normative database; RPE: retinal pigment epithelium

At the one-month follow-up in June 2024, the patient's uncorrected visual acuity had improved from 20/100 preoperatively to 20/25. Fundoscopy and spectral domain optical coherence tomography (OCT) imaging were both unremarkable (Figure 1).

## Discussion

TB dye migration into the vitreous cavity represents a rare complication of phacoemulsification that has been sporadically reported in the context of ocular trauma [3-6], uveitis [6], previous ophthalmic surgery [7], myopia [7], and pseudoexfoliation [8]. Zonular compromise could be the common underlying factor of the above risk factors, usually clinically evident as preoperative phacodonesis. Interestingly, none of the above clinical entities was part of our patient's profile. Additionally, detailed and repeat preoperative evaluation did not identify either zonular compromise or phacodonesis.

Subsequently, given our patient's history of participation in contact sports, we hypothesize that soccer-related repetitive head micro-trauma may have caused limited, subclinical impairment of the zonular integrity without evident phacodonesis, resulting in inadvertent intraoperative TB dye migration into the vitreous cavity. Soccer has been associated with chronic traumatic encephalopathy, probably the result of repetitive and cumulative micro-concussions caused by heading [9], as well as severe ocular injuries through a mechanism of direct football blunt impact, causing the deformity of the globe [10]. Interestingly, there are studies that have identified soccer as the most common cause of sports-related eye injuries, culminating in late complications (traumatic cataract, secondary glaucoma, macroscopic hyphema, and retinal detachment) caused by the rapid rise in intraocular pressure, equatorial stretching, and posterior displacement of the iris-lens diaphragm due to blunt trauma [11].

A review of the literature reveals that in all published case reports concerning TB dye posterior migration, the management was conservative, with the dye gradually resolving without additional surgical intervention [3-9]. However, possible concentration and exposure time-related toxicity both to the retinal pigment epithelium and to other retinal cells is also documented [2] and cannot be excluded, justifying our approach to offer a minimally invasive 25G pars plana vitrectomy to the patient, an established procedure with an overall safe profile, often implemented for primary symptomatic floaters [12].

Ultimately, the combined approach of phacoemulsification with premium IOL implant and pars plana vitrectomy with TB dye evacuation resulted in a satisfactory refractive and functional outcome.

## Conclusions

In the absence of identified risk factors that could compromise the zonular apparatus, we speculate that the encountered TB dye misdirection into the anterior vitreous during our patient's phacoemulsification could be possibly attributed to occult zonular micro-dehiscence, related to repetitive, soccer-associated head trauma, caused either by heading or direct football blunt impacts to the globe. These factors could have potentially compromised the delicate zonular apparatus at a subclinical level, without evident preoperative phacodonesis. In this context, intracameral TB dye could be misdirected into the anterior hyaloid, rendering the subsequent phacoemulsification steps challenging and raising concerns of possible retinal toxicity. Although soccer-related eye trauma could represent a plausible explanation of the encountered complication, the speculative nature of our interpretation and the hypothetical basis of our conclusions have to be acknowledged. Awareness of the rarely described entity of TB dye posterior migration in athletes undergoing phacoemulsification could justify surgical modifications, such as omission of TB dye use during capsulorhexis or simultaneous vitreoretinal surgery capacity to mitigate either its occurrence or its consequences. The chosen approach to this surgical scenario, either conservative or surgical, represents a dilemma and remains at the discretion of the operating surgeon.
